# Affected family members’ experience of, and coping with, aggression and violence within the context of problematic substance use: a qualitative study

**DOI:** 10.1186/s12888-017-1374-3

**Published:** 2017-06-02

**Authors:** Terence V. McCann, Dan I. Lubman, Gayelene Boardman, Mollie Flood

**Affiliations:** 1 Program of Nursing and Midwifery, Centre for Chronic Disease, College of Health and Biomedicine, Victoria University, PO Box 14428, Melbourne, VIC 8001 Australia; 2 Turning Point and Eastern Health, Melbourne, Australia; 3 Eastern Health Clinical School, Monash University, Melbourne, Australia

**Keywords:** Problematic substance use, Affected family members, Aggression, Violence, Coping, Families, Qualitative research

## Abstract

**Background:**

Families have an important role supporting a family member with problematic substance use (PSU), although this can often be challenging and confronting. Previous research has identified high rates of family aggression and violence within the context of PSU, although few studies have examined this issue from the perspective of affected family members (AFMs) supporting a member with PSU. The aims of the current study were to understand AFMs’ experience of aggression and violence while supporting a member with PSU, and to explicate the strategies they used to prevent and cope with this behaviour.

**Methods:**

Semi-structured, audio-recorded qualitative interviews were conducted with 31 AFMs from the state of Victoria in Australia. Interpretative Phenomenological Analysis was used to guide data collection and analysis.

**Results:**

Almost 70% of participants experienced PSU-related family aggression and/or violence. Two main themes and related sub-themes were abstracted from the data capturing their experiences of this behaviour and the strategies they used to try to prevent and cope in this situation. Aggression and/or violence were variable, changeable and unpredictable; and aggression and/or violence altering social interactions and family dynamics. As a consequence, it was upsetting, stressful and emotionally exhausting to AFMs. In response to this experience, and largely through trial and error, they used several direct strategies to try to prevent and cope with the behaviour; however, most continued to struggle in these circumstances. They also highlighted additional indirect measures, which, if adopted, would enhance their existing direct strategies.

**Conclusions:**

More effective primary, secondary and tertiary preventive measures are needed to address family aggression and violence within the context of PSU. More support is needed for family members affected by PSU to enable them to ‘stand up to,’ to prevent and cope effectively with this behaviour, and to increase their help-seeking and access to specialist services and support groups. More appropriate policies and social services are needed to meet the needs of AFMs.

## Background

Problematic (or problem) substance use (PSU) is increasingly prevalent in Australia and many other countries. PSU is alcohol and/or drug use that is dependent or recreational; it is not necessarily the frequency of use that is the primary ‘problem’ but the adverse consequences it has overall on the user’s life and on other family members (e.g., social, financial, psychological, physical, legal) [1]. As evidenced in this definition, harms associated with PSU are not restricted to individuals, but have significant effects on families, friends, work colleagues and even strangers [2–4]. Indeed, for affected family members (AFMs) (intimate partners, parents, siblings, offspring, other relatives, close friends) [5], who often carry the primary responsibility of supporting and advocating for the person with PSU, they not only have to manage issues of stigma and social isolation [2, 6, 7], but cope with family arguments, subsequent breakdown, abuse, aggression and violence [4]. As such, support-giving (emotional and instrumental [tangible assistance such as financial, material and practical support]) is challenging and has considerable detrimental effects on AFMs’ physical, psychological, social and financial well-being [3, 5, 6, 8–10]. These effects can undermine their ability to maintain this important support-giving role [7], and, in turn, affect adversely the person with PSU [2]. For instance, it is conservatively estimated that for every person with addiction, on average one AFM is impacted; and based on WHO prevalence data for alcohol addiction this is approximately 100 million AFMs [5, 11]. Because PSU incorporates but extends well beyond addiction, the number of AFMs is much greater.

Concerns about the impact of family aggression and violence have been growing recently [12–14], with research identifying high rates in families dealing with all forms of PSU [15–17]. The terms ‘aggression’ and ‘violence’ are often used interchangeably [18, 19], which may be due to an overlap between the terms [20, 21]. In this paper, aggression and violence is ‘any form of behaviour that is intended to injure someone physically or psychologically’ (p.6) [22]; it can take various forms (verbal, physical, emotional) and may be directed to another person or object. Alcohol and drugs are prominent contributors to intimate partner aggression and violence, with 40–60% of documented cases involving perpetrators affected by these substances [23–28]. Older women are also likely to experience family aggression and violence, often intergenerational, with the perpetrator most commonly being a son who is in a dependent relationship who has PSU [29, 30].

AFMs frequently receive inadequate support to sustain them in their support-giving role. They regularly report they are not listened to and are excluded from key treatment decisions, by service providers [5, 12]. Their involvement with services is usually minimal, unplanned and unstructured, and constrained by confidentiality issues and service requirements [31]. AFMs frequently find that supporting a member with PSU has major detrimental effects on their own well-being and their coping resources are often exhausted [5], findings similar to those focusing on families of young people with first-episode psychosis [32] and older adults with mental health disorders [33]. Indeed, England, Kennedy and Horton [34] found that 32% of families report that anger, rage, aggression and violence by the person with PSU causes them the most concern, but often they do not seek external help. AFMs also feel isolated and receive little support from other family members [35]. Families are frequently fractured because of the continuing damaging and destructive effects of the person’s PSU-related behaviour [10]. Orford et al. [5] propose a Stress-Strain-Coping-Support Model, to explain how AFMs respond to a member’s aggression and/or violence. Essentially, they use one or more of three broad approaches to cope with this behaviour: *putting up* (with the behaviour), *withdrawing* (from the person and the environment), and *standing up* (challenging the behaviour, seeking assistance from law enforcement and judicial agencies).

Overall, while some light has been shed on AFMs’ burden in supporting a person with PSU, limited research has been undertaken into their experience of aggression and violence and how they attempt to deal with this challenging behaviour. Therefore, the aims of our study were to understand AFMs’ experience of aggression and violence within the context of PSU, and to explicate the strategies they used to prevent and cope with this behaviour. The study was nested within a broader mixed methods (qualitative interviews and survey) study of the experience of AFMs supporting an adult member with PSU.

## Method

Interpretative phenomenological analysis (IPA), a hermeneutic or interpretative approach informed by the Heideggerian perspective of phenomenology [36], was adopted to guide data collection and analysis inductively. The approach requires a detailed examination of participants’ lived experience and how they interpret their personal and social world [37]. Researchers using IPA adopt a double hermeneutic in ‘trying to make sense of the participant trying to make sense of what is happening to them’ (p.3) [37]. Hence, the attempt to comprehend the participant’s perspective, necessitates interpretative activity by the researcher [38]. The social constructionist perspective, that social, situational, contextual and historical influences affect the way people perceive and experience their lives, informs IPA. In particular, the IPA interpretation of social constructionism is influenced more so by symbolic interactionism than poststructuralism, which informs most discursive psychology [37, 39]. IPA is also informed by idiography because of its focus on beginning with the individual as the unit of analysis and then progressively developing broad themes [40, 41]. Similar to most qualitative approaches, IPA requires the researcher to adopt an “emic” or insider (participant) approach when collecting and analysing data, and an “etic” or outsider (researcher) approach when applying concepts and theories to interpret the findings (in the discussion section) [42]. IPA is particularly suitable where problems are under-researched or new, are complex to understand, and where researchers strive to understand process and change [37].

### Participants and procedure

Participants were recruited through a state-wide alcohol and drug helpline service (Turning Point), Social Media (Turning Point’s Twitter account), and the Self Help Addiction Resource Centre (SHARC), in Melbourne, Australia. AFMs who contacted the helpline for assistance were given brief information about the study by helpline counsellors. Contact details of interested AFMs were forwarded, with their permission, to the researcher, while those recruited via Social Media contacted the researcher directly. Purposive sampling [43] was then used by the researcher to assess potential participants’ eligibility for the study. Inclusion criteria were: (i) AFMs of an adult, aged 18–65 years old, with PSU; and (ii) in the support-giving role for at least one year (as defined by the AFM). Exclusion criteria were: (i) previous recipient of specialist family interventions for PSU; and/or (ii) had a recent personal history of PSU or severe mental illness.

Individual, semi-structured interviews were used to examine participants’ experience of PSU-related aggression and/or violence (Table 1). All interviews took place by telephone and were audio-recorded. The study was undertaken in accordance with the ethical standards of the Declaration of Helsinki (Brazil 2013 revision). Ethics approval was obtained from Eastern Health Human Research Ethics Committee (LR59/1314). All participants provided verbal consent over the telephone (which was recorded) to participate. Issues discussed were responded to in a sensitive and supportive manner by the researcher. No participants appeared to experience distress as a result of participation in, and none withdrew from, the study.

**Table 1:** Sample of interview questions

Focus	Example of question or prompt
Overall experience of aggression and/or violence	− Could you describe an occasion(s), if any, in which the person was aggressive and/or violent? − On these occasions, what drug/alcohol was the person using? − In what way was the person aggressive and/or violent (e.g., verbal, physical, emotional, directed at a person or property)? − What things, if any, served as specific triggers to this behaviour?
Effect of aggression and/or violence on family members and others in family	− What effect, if any, has the person’s aggression and/or violence had on your health and well-being and that of other family members? − What effect, if any, has the person’s behaviour on the way your family is structured and operates, and how family members support each other?
Preventing and coping with aggression and/or violence	− What things, if any, do you do to help prevent and cope with the person’s aggression and/or violence? − Is there anything you would do doing differently, to help you cope with this behaviour? − Are there any other things that could help you prevent and cope with this behaviour?

### Data analyses

Interviews were transcribed verbatim and anonymised. Transcripts were read and re-read to obtain a broad appreciation of AFMs’ experience of aggression and/or violence and coping. Coding was undertaken using NVivo [44]. Initially, in-vivo codes were used, which prevents researchers from superimposing pre-existing beliefs or theories on data [45]. Then, codes were clustered into provisional themes and sub-themes. Simultaneously, data reduction occurred with provisional themes omitted if insufficiently grounded in the data. A more intense analysis then produced a fine-tuning of themes and abstraction to a higher level [46]. This iterative and inductive process was continued throughout the analysis to ensure themes were adequately saturated [39] with a ‘thick’ description of the data and no additional data emerged to support the themes. This process also established the number of study participants, a key aspect of the rigour of qualitative research in determining sample size [47]. A semantic level of analysis was carried out, progressing from description and summary in the results section, to interpretation in the discussion section [48].

### Reflexivity

Many qualitative researchers believe that it is impossible to obtain absolute knowledge of reality — in the present study, using IPA to explicate AFMs’ experience of aggression and violence — because participant recruitment, data collection and analyses risk being tinged by researchers’ prior experiences, assumptions, values, expectations and priorities [37, 49, 50]. This criticism can be overcome by being reflexive or self-aware [37, 45, 51], or what Van Maanen (1988, pp.73–100) referred to as adopting a “confessional” style, at several levels. First, researchers should make explicit the theoretical perspective they are adopting — in this instance, IPA. Second, in order to avoid or minimise the likelihood of introducing bias into the study, it is essential to examine at the outset the researchers’ preconceived assumptions and expectations about the phenomenon under study (in this case, aggression and violence). In this study, the researchers were aware that aggression and violence occurred within the context of substance use in families but did not hold preconceived beliefs about attributing blame for this behaviour to AFMs and/or people with PSU. Third, it is important to reflect on these assumptions and expectations throughout all stages of the study, to be self-questioning and self-aware [50]. Fourth, the researcher should explain and reflect upon the research methods used in the study and the context in which it was undertaken. In this study, all interviews were conducted by a female researcher with a background in conducting research in the substance use field, and a semi-structured interview guide was used to guide the interview process. Furthermore, initial thematic analysis was undertaken by TMcC, followed by an independent review of the process by DL and GB, which enhanced the rigour of the study [43]. Differences in coding and theme identification were resolved through discussion until consensus was achieved.

## Results

Thirty-one AFMs participated in the interviews, conducted between January and December 2015. Participants comprised parents (*n* = 14), partners (*n* = 13) and siblings (*n* = 4) from the state of Victoria, in Australia. Most participants were female (81%) and all partners were female. Their mean age was 47 years (ranging from 26 to 68 years), and almost 70% experienced family aggression and/or violence. The mean duration of time participants had been in the support-giving role was 8.05 years (ranging from 1 to 20 years). The main form of PSU in their families was alcohol (*n* = 14, 45%), a combination of alcohol and drugs (*n* = 14, 45%), and drugs alone (*n* = 3, 10%). Principal drugs of concern were methamphetamine (especially crystal methamphetamine) (*n* = 5, 16%), cannabis (*n* = 4, 13%), and heroin (*n* = 4, 13%). Polydrug use was highlighted as a concern by several participants (*n* = 4, 13%).

Two main themes, and related sub-themes, were abstracted from the data: *aggression and violence as stressful and emotionally exhausting,* and *struggling to prevent and cope with aggression and violence.*

### Aggression and violence as stressful and emotionally exhausting

In this theme, the experience of being an AFM of an individual with PSU who was aggressive and/or violent was upsetting, stressful and emotionally exhausting. Participants felt overwhelmed, confused, anxious and frustrated as they faced the daily challenge of supporting a person whose mood, attitude and behaviour were unpredictable and frequently adversarial. The person’s apparent lack of awareness of the impact of this behaviour on the family exacerbated participants’ sense of frustration and futility. Despite this significant challenge, they affirmed their commitment to persevere supporting the individual.

*It’s a war zone, because our aim is to get him through this somehow, some way.* (Interviewee 30, mother)

*He's a much loved member of the family and I think if we had not supported him emotionally, then he might well have been dead or been in a very bad place.* (Interviewee 9, father)

*The worst part for me was not knowing that person anymore and knowing he’d done things that the brother that I once knew would never have done.* (Interviewee 5, brother)

AFMs felt anxious and physically and emotionally exhausted because of the person’s aggression and/or violence. For some, their anxiety had an adverse effect on their ability to cope with routine activities.

*Everything I did was affected, because I was so full of anxiety, I just couldn’t cope with everyday tasks.* (Interviewee 5, brother)

*I try to be supportive, but it really affects my mental stability and physical exhaustion.* (Interviewee 26, female partner)

The experience of aggression and/or violence was particularly challenging, as AFMs sought to maintain a reasonable relationship with the person. Two sub-themes were abstracted from the data, reflecting their experience of this challenging behaviour as stressful and emotionally exhausting: *violence as variable, changeable and unpredictable,* and *aggression and/or violence altering social interactions and family dynamics.*

### Aggression and/ violence as variable, changeable and unpredictable

In this sub-theme, AFMs regarded their aggression and/or violence experience as a major concern. The behaviour was long-term, variable, changeable over time and unpredictable.

*He starts swearing a lot when he talks. He’s very quick to anger, unreasonable and can be explosive.* (Interviewee 20, female partner)

*Very angry, can be violent, screams at you … grumpy is not a good enough word.* (*Interviewee* 4, mother)

*He was quite violent towards me, my wife, the house.* (*Interviewee* 21, father)

Participants distinguished between aggression and violence. Verbal and emotional aggression was characterised by shouting, insults, criticism and/or harassment. The behaviour usually constituted arguments, telling lies and manipulation, whereby the person tried to force the AFM to accept their view, or force them to comply with their wishes. Participants also reported instances of being ignored or blamed unreasonably for things by the person.

*We argue a lot; I guess that's around lying and manipulation and things like that.* (*Interviewee* 10, female partner)

*They lie and then they blame you … it's awful and very hard.* (Interviewee 14, female partner)

Emotional aggression extended to limiting AFMs’ freedom of movement, reducing their lifestyle because of the person’s PSU-affected misperception of things. It also had adverse consequences for family structures, in particular, for future grand-parenting roles.

*I’m having to change my lifestyle, because of his misconception of everything.* (Interviewee 19, father)

*Knowing her father’s an alcoholic; well, I know we won’t be grandparents who look after the child.* (Interviewee 1, female partner)

Participants experienced physical violence over time, such as pushing, punching and biting. They reported threats of physical violence using weapons; fortunately, threats did not materialise into actual use of weapons.

*There has been push and shove and he has hit the wall.* (Interviewee 30, mother)

*Violent, smashing things, just really awful to be around, threaten a lot of things.* (Interviewee 5, sister)

*While I was pregnant, my sister threatened to stab me in the stomach and kill my baby.* (Interviewee 27, sister)

Several AFMs commented about experiences of the person deliberately, and often seriously, causing property damage. This form of violent behaviour ranged from punching holes in walls, breaking household items, to crashing cars.

*I've got holes in my house (walls) everywhere.* (Interviewee 27, sister)

*He's already wrecked three cars of mine. I've got a brand new one now, so I've hidden the keys.* (Interviewee 18, mother)

Sometimes, aggression and/or violent episodes were unpredictable and triggered by something seemingly innocuous. In some cases, participants reported that they could not identify a trigger for the behaviour, other than substance use.

*It can be really wonderful one minute and then he can just change like that.* (Interviewee 18, mother)

*He hits a certain point and goes downhill and becomes irritable … it might be about something at work, it might be about something I've said or done, as small or trivial or whatever as it is.* (Interviewee 26, female partner)

### Aggression and/or violence altering social interactions and family dynamics

In this sub-theme, the person’s aggression and/or violence had a direct adverse effect on social interactions with AFMs’ friends and family dynamics. Because of the unpredictable and undesirable nature of the behaviour, it contributed to their social isolation from friends, reducing their access to this potential form of support.

*I don't feel comfortable inviting people over to my house, because I don't know what he's going to be like.* (Interviewee 10, female partner)

Aggression and/or violence also impacted unfavourably on family dynamics. At times, children assumed a caretaker or protector role consciously or sub-consciously, and frequently played different roles before, during and after an episode of this behaviour. Their roles changed from being ‘passive’ witnesses, to assuming active roles to protect themselves or other AFMs from it, to subduing the frequency and severity of outbursts, of the behaviour.

*I think my husband doesn't take us on as much because [my son] will step in and protect us now physically.* (Interviewee 11, female partner)

While these role changes provided new support to AFMs, they could create additional tensions between family members, and with the young person who had to adapt to the change in his/her family status. Even though mother participants appreciated the support they received from their growing children, this came at a cost to some adult children or siblings missing out on having ‘normal’ age-appropriate parent or sibling relationships. It could also lead to role reversal situations, whereby a son or daughter would become the main support person to the parent with PSU.

*I feel sad, the profound effect it's had on the children these years.* (Interviewee 11, female partner)

*You don’t have a normal mother-daughter relationship … I’ve been the mother and she’s been the daughter.* (Interviewee 15, daughter)

### Struggling to prevent and cope with aggression and/or violence

In response to aggression and/or violence being stressful and emotionally exhausting, and largely through trial and error, AFMs identified several direct measures they used to try to prevent and cope in these circumstances; however, most continued to struggle to deal with the behaviour. They also highlighted additional indirect strategies, which if implemented, would enhance the existing strategies used to cope with aggression and/or violence.

#### Strategies used to prevent and cope with aggression and/or violence

Most participants developed several strategies to try to prevent and cope with the behaviour. Four main prevention- and coping-oriented strategies were developed.

##### Maintaining constant vigilance to avoid triggering aggression and/or violence

Participants identified the need to maintain constant vigilance to recognise early signs of aggression and/or violence and avoid triggering an episode. This entailed treading carefully, being cautious about words or actions that might trigger an episode of the behaviour, a fragile situation they equated to ‘walking on eggshells’. Constant vigilance left AFMs resigned to their circumstances, as they tried to cope with the person’s behaviour, but without much hope of change.

*That’s the hardest … always thinking about what I have to say or how I have to say something.* (Interviewee 1, female partner)

*I've come to realise that the signals and the triggers that are coming and I'll avoid them. I don’t want to have to live this way, having to watch what I say in case it ends up in an argument …. I tread carefully and avoid an argument, because a word can trigger an argument.* (Interviewee 19, father)

*When he’s drinking, I am walking on eggshells because he gets so angry so fast.* (Interviewee 20, female partner)

##### Curtailing social activities

Some participants commented that aggression and/or violence was associated with engagement in social activities with the person with PSU. This situation made it difficult for AFMs to plan ahead and enjoy social activities, as they could not assume the person would behave appropriately. As a consequence, intimate partners in particular sometimes resorted to curtailing social activities to reduce the risk of the behaviour being triggered. While curtailing social activities had some benefits, it also reinforced AFMs’ isolation and limited their access to informal support.

*There's always a fight and drama and we can't just have a weekend somewhere without a memory of him fighting.* (Interviewee 26, female partner)

##### Disengaging from the person when PSU occurs

Once they realised the person was taking substances, some participants adopted a disengagement coping strategy to protect themselves from aggression and/or violence and the associated stress and anxiety. Disengagement ranged from distancing oneself physically from the person, not engaging the person in conversation, and in extreme circumstances, leaving the family home.

*These days I do a lot of disengaging, because rather than stick around when he's very drunk, I will just go off and do something else.* (Interviewee 3, female partner)

*I've learnt not to talk to him and also encouraged the kids not to talk to him when he's drinking.* (Interviewee 24, female partner)

*I had to move him out of the house for a year when he started threatening my wife, which was really hard for all of us here.* (Interviewee 9, father)

##### Seeking help from law enforcement and judicial agencies

When the person’s aggressive and violent behaviour exceeded AFMs’ routine coping measures, some sought help from law enforcement and legal agencies. In practice, this entailed seeking direct help from the police and/or obtaining a Family Violence Intervention Order [52] from a Magistrates’ Court to protect their family. Female AFMs were more likely to call the police, while fathers reported they took this initiative on some occasions, seeking to protect their partners or other children from the person’s behaviour. However, if the Family Order resulted in the person with PSU being excluded from the family home to prevent the behaviour, the exclusion process also caused distress and guilt for parents in particular.

*He was really violent, really aggressive. I had to call the police a few times.* (Interviewee 5, sister)

*Yeah, there have been situations where we have to call police and have her restrained.* (Interviewee 23, sister)

*There were a couple of intervention orders put out over the time, and criminal charges through violence.* (Interviewee 21, father)

Only one female intimate partner reported currently having an active Intervention Order against her male partner. This permitted him to live with her, conditional on him not being aggressive and violent towards her. The police, who had witnessed the person punching the woman, instigated the Order.

#### Additional indirect strategies to enhance prevention and coping with aggression and violence

Participants identified two additional indirect strategies, which, if adopted, would enhance their existing direct measures to prevent and cope with aggression and violence.

##### Increasing access to specialist support services

Even though most participants were recruited through an alcohol and drug helpline service in this study — primarily because of a crisis situation in their families — only a few indicated that they had sought other forms of help about supporting the person’s PSU. However, none gave specific examples of formal help seeking to enhance the existing strategies they used to prevent and cope with the behaviour. Other participants used the Internet to find out how to cope in this situation, but were concerned about, what they perceived as, inconsistent and confusing information. In essence, this highlighted the need for information to be evidence-based, relevant and easily accessible to AFMs.

*My boss is a psychologist and she has a daughter who went through all of this. She gives me some books and research things and stuff. So I read a lot of that, but then I also read online. But it's such conflicting information as well, which I find quite tricky.* (Interviewee 27, sister)

*To be honest, I looked for some kind of online support service and I just couldn’t find one that I felt was relevant to my situation.* (Interviewee 10, female partner)

Primarily because of lack of engagement with alcohol and drug services, participants had not received education on aggression and violence coping strategies from professionals. A lack of evidence-based and specialist-provided knowledge on how to cope effectively in this situation was highlighted. They were also unsure how to access evidence-based information or specialist alcohol and drug treatment services that might help them prevent and cope with the behaviour.

*I've never really been aware of what support or if anyone could help us with all the things that we're going through with it.* (Interviewee 11, female partner)

*Learning some strategies to deal with the behaviours. Not to get involved with the arguments. To walk away when the alcoholic’s ranting and raving.* (Interviewee 15, daughter)

*I need somebody to sit down and spend the time needed with both of us.* (Interviewee 26, female partner)

##### Sharing aggression and violence experiences with other families

Even though most participants were recruited through a state-wide helpline, they were often unaware of the existence of PSU support groups for families. Despite this lack of awareness, they felt they would benefit from sharing their experience of aggression and violence with other AFMs in similar circumstances, to enable them to obtain mutual support and develop appropriate and effective measures to prevent and cope with this behaviour.

*Some kind of group for supporting family members would be nice … maybe people have other things that they can suggest and stuff like that.* (Interviewee 3, female partner)

*I was hoping there was a meeting with other people going through the same thing I’d been going through, just to hear their stories and how other people on average respond to situations like this.* (Interviewee 16, female partner)

## Discussion

In this exploratory study, we provide a rich understanding of the under-researched phenomenon of AFMs’ experience of aggression and violence from a member with PSU, with an emphasis on comprehending the measures they took to try to prevent and cope in these circumstances. Two main themes were abstracted from the data depicting their experience of aggression and violence as stressful and emotionally exhausting and struggling to prevent and cope with this behaviour. In the first theme, *aggression and violence as stressful and emotionally exhausting,* AFMs frequently felt overwhelmed, confused, anxious and frustrated with the person’s behaviour. These findings are consistent with those of other studies, that supporting an individual with PSU, especially when the person is aggressive and violent, has major adverse effects on AFMs’ well-being and their coping resources are frequently depleted [5, 10, 34]. In the present study, participants also commented that the behaviour was variable and changeable, inconsistent and unpredictable. This finding was also reported in a United States study of women subjected to intimate partner aggression and violence, where the behaviour of people with varying levels of PSU was often inconsistent and unpredictable, and it could escalate rapidly before those around them could prevent the onset of, or prepare for, an episode [53]. In the present study, some participants received varying forms of informal support from other family members, including their parents, adult children and siblings. This finding, about families being supportive, contrasts with that of another study where the AFM giving the most care received minimal support from other family members [35].

In the current study, family support came at a cost to some adult children or siblings missing out ‘normal’ age-appropriate parental or sibling relationships. The concept of parentification or parental-child role reversal occurs in this situation. Parentification in the family is “a functional and/or emotional role reversal in which the child sacrifices his or her own needs for attention, comfort, and guidance in order to accommodate and care for logistical or emotional needs of the parent” (p. 5) [54]. The concept can take two forms with the child; it may be instrumental (e.g., takes responsibility for cooking, washing, household finances) and/or emotional (e.g., protects the parent when another family member is aggressive or violent, takes on the role of being a friend to the parent). The duration of the parentification role is an important consideration. The role may be short-term (e.g., a parent is unwell for a short period of time) or long-term (e.g., a parent with PSU). In the short-term, the role can be ‘healthy’ or ‘adaptive’ to the child, temporarily taking on an adult’s role and obtaining an insight into adult responsibilities. In the long-term, the role can be ‘pathological’ or ‘unhealthy’, where the child works beyond their capacity, curtailing the opportunity to engage in normal childhood activities and experiences [54, 55]. Data from the present study suggest that the parentification role adopted by some of the adolescent children of family members with PSU was long-term and unhealthy to their emotional development.

In the second theme, *struggling to prevent and cope with aggression and violence,* AFMs used trial and error to develop several direct measures to try to prevent and cope in this situation; however, most continued to find it difficult to accept the behaviour and respond appropriately in these circumstances. They also identified additional indirect approaches to enhance their existing prevention and coping strategies. Generally, their coping strategies could be equated with the Orford et al. [5] Stress-Strain-Coping-Support Model, where AFMs used one or more of the three approaches to coping with the person’s aggression and/or violence: *putting up*, *withdrawing*, and *standing up*. It is evident, however, that AFMs had little or no access to specialist support and evidence-based information to assist them in this situation. An indication that these strategies were insufficient is that some sought assistance from law enforcement and legal agencies. While this may be interpreted as an appropriate ‘standing up’ response in this circumstance, it also highlights the limitations of their other routine coping strategies. The findings about limited access to specialist services and lack of evidence-based information are consistent with those from other studies, that families frequently do not engage in formal external help-seeking [35], and such engagement is often negligible, unplanned and unstructured [31]. On a broader level, lack of help-seeking may be attributable to AFMs feeling disempowered as a consequence of their close contact with family members with PSU, who themselves are increasingly disempowered as a result of their substance use problems [56], poverty and social exclusion [57]. In addition, and similar to other studies [5], the majority of AFMs in the present study were women (81%). Hence, it could be interpreted that some women were doubly disempowered: because of their close proximity to the family member with PSU and, for intimate partners in particular, through living with men with a traditional gender-role inclination [5].

One common explanation in the present study for AFMs’ lack of help-seeking from specialist services and support groups is they were unaware of the existence of these services or were only recently aware of their existence (all were recruited through telephone support or web/social media alcohol and drug services). This situation may be attributable to several service-related influences, including many not being funded to provide support to families; actively excluding families from accessing and participating [12]; lack of knowledge about, insensitivity to, and empathy for, families; too few providing psychosocial interventions for families; and the need for a stronger focus on families [5]. Moreover, AFMs in the present study were concerned about, what could be regarded as, a lack of relevant, accessible and evidence-based information to assist them generally about PSU and specifically about aggression and violence. Therefore, lack of access to and support from specialist services, and lack of evidence-based information compounded their unfavourable experience of this behaviour and undermined their capacity to prevent and cope in these circumstances. These findings are supported by a Canadian study that highlighted the need for improved AFM access to specialist services, where they are not judged, are listened to and treated with respect [12]. It could also be interpreted that lack of access, support and evidenced based information in the present study could, implicitly, infer a degree of blame on AFMs for their situation. Indeed, assisting AFMs to support the person to seek treatment is a key strategy for dealing with PSU and family aggression and violence, as both need to be treated simultaneously to be effective [14, 58]. Furthermore, there is a need for support for AFMs to extend beyond the immediacy of coping with family members’ aggression and violence, and other aspects of their behaviour, to enabling AFMs to become independent of their support-giving role and to re-focus on meeting their own rights and needs [5]. Within the context of the Orford et al. [5] Stress-Strain-Coping-Support Model, better help-seeking, access, informal and formal support as well as re-focusing on meeting their own needs may equip AFMs more so to ‘stand up to’ (engaged coping) and prevent episodes of aggression and violence instead of having to ‘put up with’ or ‘withdraw from’ this behaviour.

### Limitations

The study has three main limitations. It is a qualitative study, and findings are context bound to the participants and settings in which recruitment occurred. Although generalisability is not a prerequisite of qualitative research [59], findings are verifiable [60, 61] and provide an important guide for AFMs and specialist service providers in other PSU contexts. Recruitment through alcohol and drug related services may have produced an atypical sample of partially engaged AFMs. Future research might benefit from recruiting those not engaged with these services. Finally, future research should aim to recruit more men as their experience as AFMs may be somewhat different to that of women participants.

## Conclusions

Our study presents insightful findings into the under-researched area of AFMs’ experience of, and the measures they took to prevent and cope with, aggression and violence in the context of supporting a member with PSU. The behaviour is stressful and emotionally draining, and participants used a range of measure to prevent and cope in this situation; however, most continued to struggle to cope in these circumstances. The findings add to knowledge of how we can best support AFMs in different situations to ‘stand up to’ this behaviour. The findings also have implications for the introduction of more effective primary, secondary and tertiary preventive measures to tackle family aggression and violence. AFMs need better access to specialist services and support groups as well as evidence-based strategies to deal with this behaviour. At the same time, clinicians in alcohol and drug use services need to be more open to, and supportive of, AFMs. The findings also have broader implications for the development of appropriate policies and social services to meet the needs of AFMs of family members who are aggressive and violent in particular, and for PSU in general, and for supporting AFMs to maintain (or re-gain) their own independence, rights and needs. Linked to this, is the need for hypothesis generation for future studies to develop, implement and evaluate practical measures to assist AFMs to prevent and respond effectively (or ‘stand up to’) to aggression and violence.

## Abbreviations

AFMs: affected family members
PSU: problematic substance use
